# A simplified method to isolate rice mitochondria

**DOI:** 10.1186/s13007-020-00690-6

**Published:** 2020-11-04

**Authors:** Yanghong Xu, Xiaoyi Li, Jishuai Huang, Leilei Peng, Dinghui Luo, Qiannan Zhang, Zhiwu Dan, Haijun Xiao, Fang Yang, Jun Hu

**Affiliations:** 1 State Key Laboratory of Hybrid Rice, Engineering Research Center for Plant Biotechnology and Germplasm Utilization of Ministry of Education, College of Life Sciences, Wuhan University, Wuhan, 430072 Hubei China; 2 College of Life Sciences, Sichuan University, Chengdu, 610065 Sichuan China

**Keywords:** Mitochondria isolation, Protoplast, Rice

## Abstract

**Background:**

Mitochondria play critical roles in plant growth, development and stress tolerance. Numerous researchers have carried out studies on the plant mitochondrial genome structure, mitochondrial metabolism and nuclear-cytoplasmic interactions. However, classical plant mitochondria extraction methods are time-consuming and consist of a complicated ultracentrifugation procedure with expensive reagents. To develop a more rapid and convenient method for the isolation of plant mitochondria, in this study, we established a simplified method to isolate rice mitochondria efficiently for subsequent studies.

**Results:**

To isolate rice mitochondria, the cell wall was first disrupted by enzymolysis to obtain the protoplast, which is similar to animal mitochondria. Rice mitochondria were then isolated with a modified method based on the animal mitochondria isolation protocol. The extracted mitochondria were next assessed according to DNA and protein levels to rule out contamination by the nucleus and chloroplasts. Furthermore, we examined the physiological status and characteristics of the isolated mitochondria, including the integrity of mitochondria, the mitochondrial membrane potential, and the activity of inner membrane complexes. Our results demonstrated that the extracted mitochondria remained intact for use in subsequent studies.

**Conclusion:**

The combination of plant protoplast isolation and animal mitochondria extraction methods facilitates the extraction of plant mitochondria without ultracentrifugation. Consequently, this improved method is cheap and time-saving with good operability and can be broadly applied in studies on plant mitochondria.

## Background

Mitochondria were first discovered in 1850 and are considered to have evolved from the engulfment of an α-proteobacterium by a precursor of the modern eukaryotic cell [1]. The differences between plant mitochondria and animal mitochondria are small regarding their size and function since both of them evolved from the same microorganism [2, 3], but the plant mitochondrial genome can be 100 times larger than those of animals [4, 5].

Mitochondria produce more than 90% of the cellular energy (ATP) required for an organism’s growth, reproduction, and maintenance [6]. In plants, dysfunction of the mitochondria always leads to retardation of plant growth, hypersensitivity to disease, embryo lethality, pollen abortion and other disorders [7–9]. The mitochondrion is also vital to the mammal, as deficiency in mitochondrial function results in many serious diseases, such as Alzheimer’s, Parkinson’s, and Huntington’s disease [10–12]. Recently, a growing number of researchers have focused on studies of the mitochondria to explore an efficient therapeutic approach to deal with existing and emerging mitochondrial diseases [13, 14]. A large number of studies have revealed that the reason for mitochondrial deficiency might be the incompatibility between mitochondria and the nucleus or mutations in the mitochondrial genome. Generally, these dysfunctions result from disorders of transcriptional, post-transcriptional and translational regulation. Many groups have performed plant mitochondrial omics studies, and the interaction between the nucleus and the mitochondrion has contributed to speciation and cytoplasmic male sterility, which could be applied to explore crop hybrid vigor [15–22].

The classic method of rice mitochondria isolation was reported a dozen years ago and has been broadly applied in many labs, including ours [23]. Nevertheless, there are still some flaws in this method. First, it needed a large amount of plant material, since there is a great loss during the extraction process. Second, it also requires heavy labor to grind the materials. Third, the preparation of the Percoll gradient solution, an expensive reagent, is also laborious and time-consuming. More importantly, a high-speed centrifuge and an ultra-speed centrifuge are indispensable. All these requirements prohibit most labs from isolating plant mitochondria and conducting mitochondria-associated studies.

In this study, we established an improved method to extract rice mitochondria. In this procedure, we excluded the heavy labor, the expensive equipment and reagents, and reduced the requirement for the enormous amount of material. To confirm the purity of the isolated plant mitochondria, we assessed the extracts at the DNA and protein levels. The results of the proteinase digestion assay and the electron scan microscopy observation also confirmed the intact structure of the mitochondria. The mitochondrial membrane potential and functional activity of the inner membrane electron transport chain (ETC) complex were checked to assure the suitability of the mitochondria for subsequent studies.

## Results

### Workflow of the mitochondria isolation

**Fig. 1:**
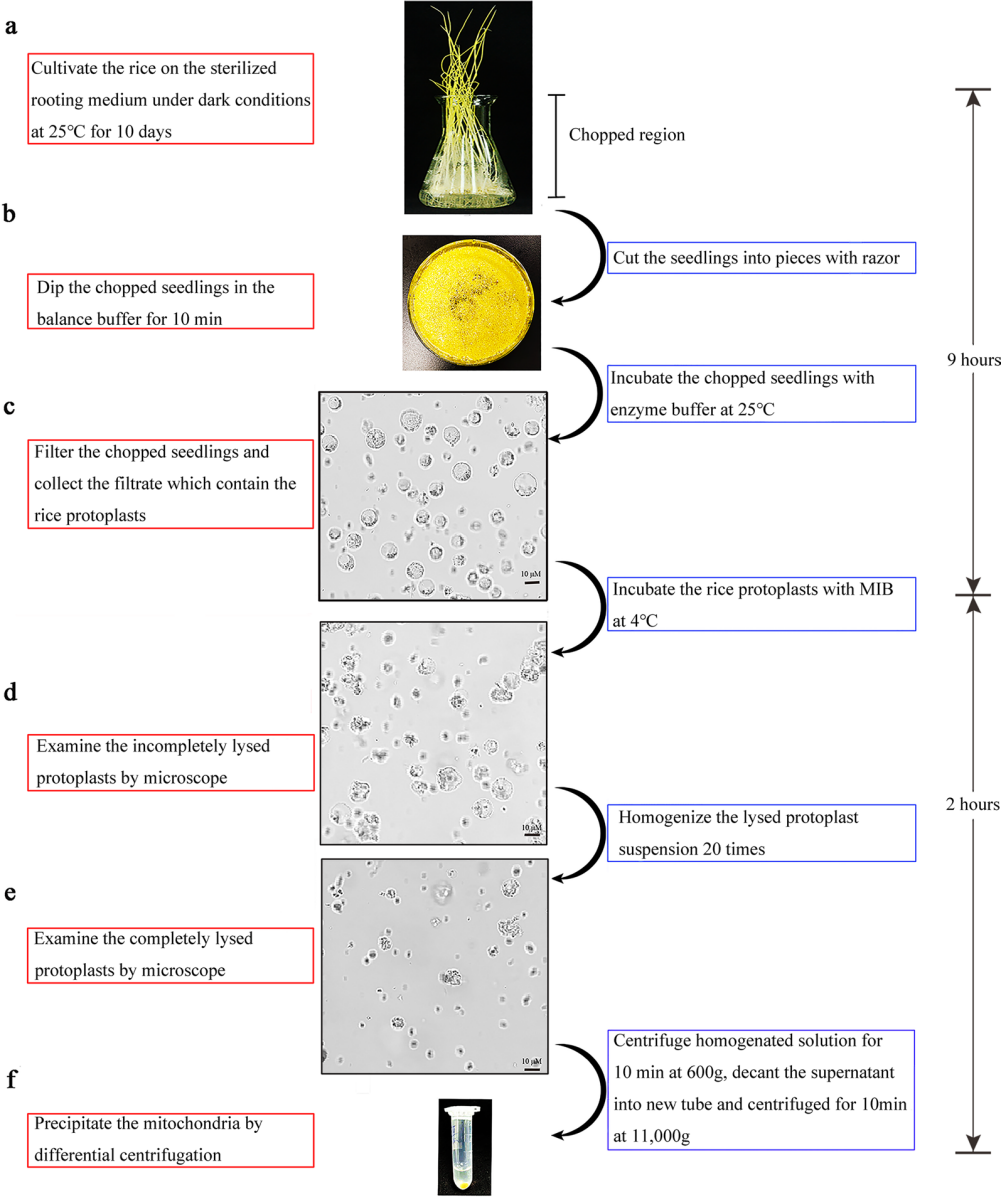
Procedure for mitochondria isolation from rice seedlings. The procedure contains two parts: the protoplasts extraction (**a**–**c**) and the mitochondria isolation from protoplasts (**d**–**f**). The reverse double arrows show the times consumed by each part. MIB: mitochondria isolation buffer. Bars, 10 μm

In this study, we improved the mitochondria extraction method by combining the traditional method of plant protoplast isolation and the mammalian mitochondria extraction protocol with slight modifications [24, 25] (Fig. 1).

First, the rice seeds were cultured in 1/2 MS medium at 25 ℃ in a dark environment for 10 days to avoid bacterial contamination. Next the 10-day-old seedlings were cut into ~ 0.5 mm pieces, which were dipped in hyperosmotic buffer for 10 min, followed by further incubation with enzyme buffer for 5 h at 28 ℃ or overnight at 25 ℃ [26]. The results suggested that the treatment overnight at 25 ℃ could yield more viable protoplasts (Table 1). Subsequently, the protoplasts were further collected by centrifugation at 600 g for 5 min and resuspended gently in mitochondrial isolation buffer (MIB). The suspension was incubated at 4 ℃ for 1 h, and the protein inhibitor PMSF was added to a final concentration of 1 mM to prevent degradation of the mitochondria by cytoplasmic proteinases. To obtain the highest yield of the mitochondria without destroying its integrity, the optimal amount of sample to homogenize was determined. Finally, the sample was centrifuged at 600 g for 10 min, the supernatant was decanted into another tube, and the mitochondria were collected by centrifugation at 11,000 g for 10 min. By this procedure, we obtained approximately 10 mg of mitochondria from 12 g of seedlings.

**Table 1:** Yields of protoplasts and mitochondria from seedlings treated with enzyme buffer under different incubation temperatures and times

Dataset	25 ℃			28 ℃		
	5 h	8 h	12 h	5 h	8 h	12 h
Protoplasts (Number)	3.75 × 10^7^ (0.75)	7.55 × 10^7^ (1.65)	5.10 × 10^7^ (0.40)	5.40 × 10^7^ (0.54)	6.00 × 10^7^ (1.20)	1.65 × 10^7^ (0.54)
Mitochondria (mg)	7.00 (3.12)	12.00 (6.54)	8.50 (5.68)	9.00 (3.00)	9.00 (0.00)	4.85 (3.76)

The numerical values in each cell represent the mean and standard deviation (in parentheses)

### The purity of the mitochondria

**Fig. 2:**
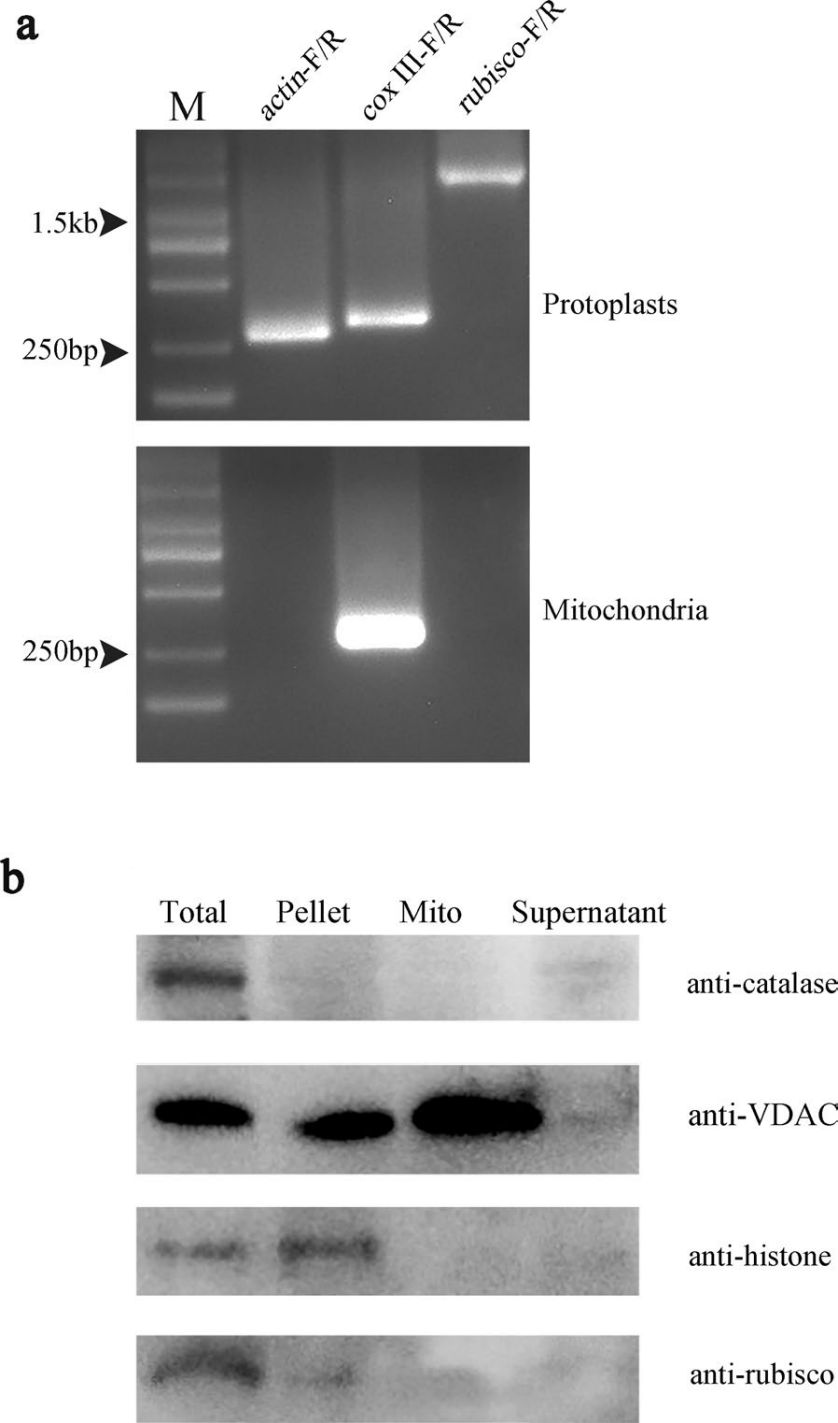
Determination of mitochondrial purity. **a** The existence of the nuclear genome, chloroplast genome and the mitochondrial genome was examined by *actin*, *rubisco* and *cox III*, respectively. M, DNA 2000 marker. **b** Four components of cells were collected for this experiment. The total lane indicates the protoplasts, the pellet lane indicates lysates from S9 (as outlined in the Sect. “Materials and methods”), the mito lane indicates the mitochondria and the supernatant lane indicates the cytoplasmic, which was derived from the supernatant in S10 (as outlined in the Sect. “Materials and methods”). Rubisco, histone, VDAC and catalase were examined as the marker proteins of the chloroplast, nucleus, mitochondria and peroxisome, respectively

To assess the purity of our isolated mitochondria, we first employed a PCR assay to assess at the DNA level. *COX III*, *rubisco* and *actin* were selected as the mitochondrial, chloroplast and nuclear marker genes, respectively. No *actin* and *rubisco* PCR products were observed even after 35 cycles of amplification, indicating that DNA isolated from purified mitochondria were free from the nuclear and chloroplast DNA contamination (Fig. 2a).

Furthermore, several specific commercial antibodies, anti-histone, anti-rubisco, anti-catalase and anti-VDAC (voltage-dependent anion channel) were applied to estimate contamination from the nucleus, chloroplast, cytoplasm and mitochondrion, respectively. Three types of components were further analyzed: the supernatants that contained the cytoplasmic substance in step 10 (S10, outlined in the Sect. “Materials and methods”) were collected, and the precipitates in step 9 (S9, outlined in the Sect. “Materials and methods”) that contained the cell lysates and the intact protoplasts were retained. The results showed that all of the cell organelle maker proteins could be detected in the total cells, indicating that the protoplast was intact (Fig. 2b). In the mitochondria fraction, only the marker protein VDAC could be detected, indicating that the mitochondria were solidly enriched. No evidence of peroxisomes, rubisco or histones was detected in the mitochondria fraction, suggesting that most of organelles were removed (Fig. 2). All these results demonstrated that the mitochondria had been isolated with a high purity.

### Determination of the mitochondrial integrity

**Fig. 3:**
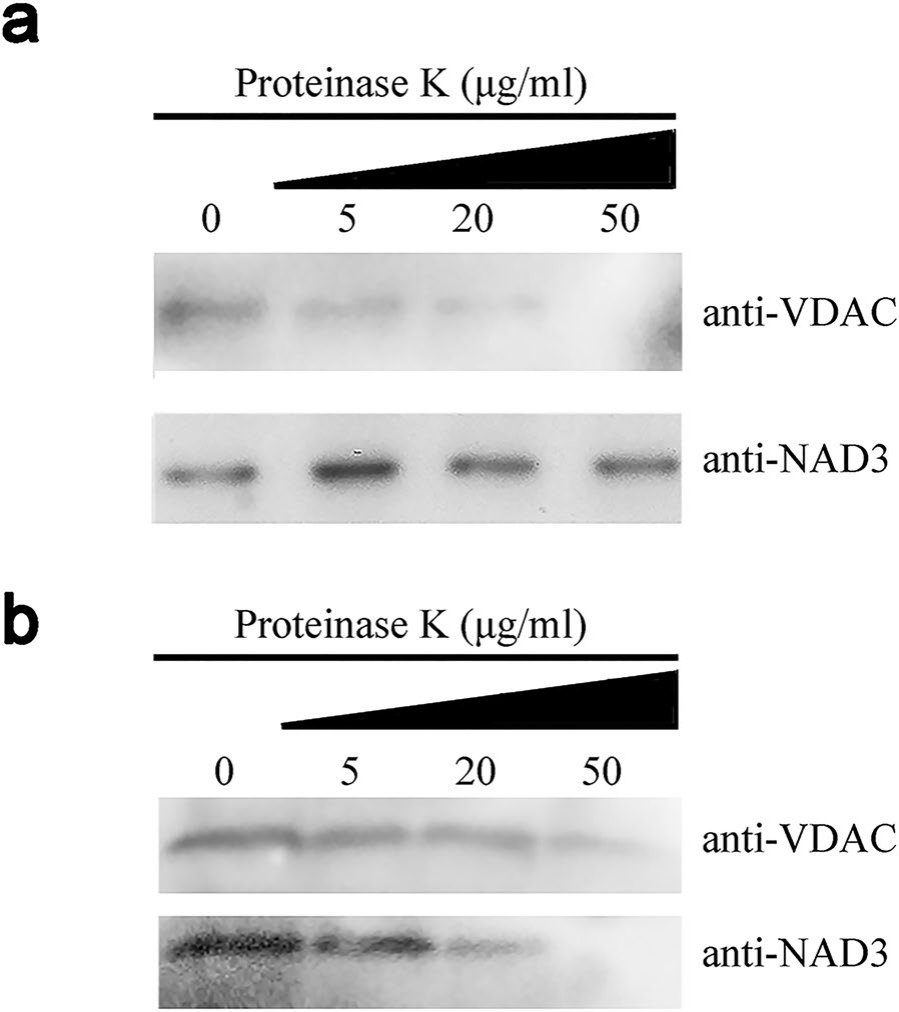
Assessment of mitochondrial integrity. Proteinase K was applied to digest the membrane proteins of untreated mitochondria in panel **a,** and lysis buffer was used to treat the mitochondria in panel (**b**). Antibodies against VDAC and NAD3, which represent the marker proteins of the mitochondrial outer membrane and inner membrane, respectively, were used for detection

To determine whether the extracted mitochondria were intact, we examined the integrity of the mitochondrial membrane via proteinase K treatment. The inner membrane proteins are more resistant against proteinase K than those located on the outer membrane in the intact mitochondrion, while in the fractured mitochondrion, the two types of proteins show identical degradation patterns when exposed to proteinase K. The result showed that the outer membrane protein VDAC was degraded under the proteinase treatment, while no degradation occurred for the inner membrane protein NADH dehydrogenase subunit 3 (NAD3) with the increasing concentration of proteinase K (Fig. 3a). In contrast, both VDAC and NAD3 exhibited identical degradation patterns when the mitochondria were treated with proteinase K after resuspension in full lysis buffer (FLB), suggesting that the membranes were destroyed completely (Fig. 3b). We also used thermolysin to assess the integrity of the mitochondrial membrane. In the chloroplasts, thermolysin can digest the outer membrane peptide but it cannot penetrate into the intermembrane space [27]. Therefore, we proposed that thermolysin would have a similar biological effect on the mitochondrial outer membrane. Under treatment with an increasing thermolysin concentration, we observed the continuous degradation of VDAC, whereas the inner membrane marker protein COXII remained stable (Additional file 1: Fig. S1). This distinct degradation pattern implied that our extracted mitochondria were intact.

**Fig. 4:**
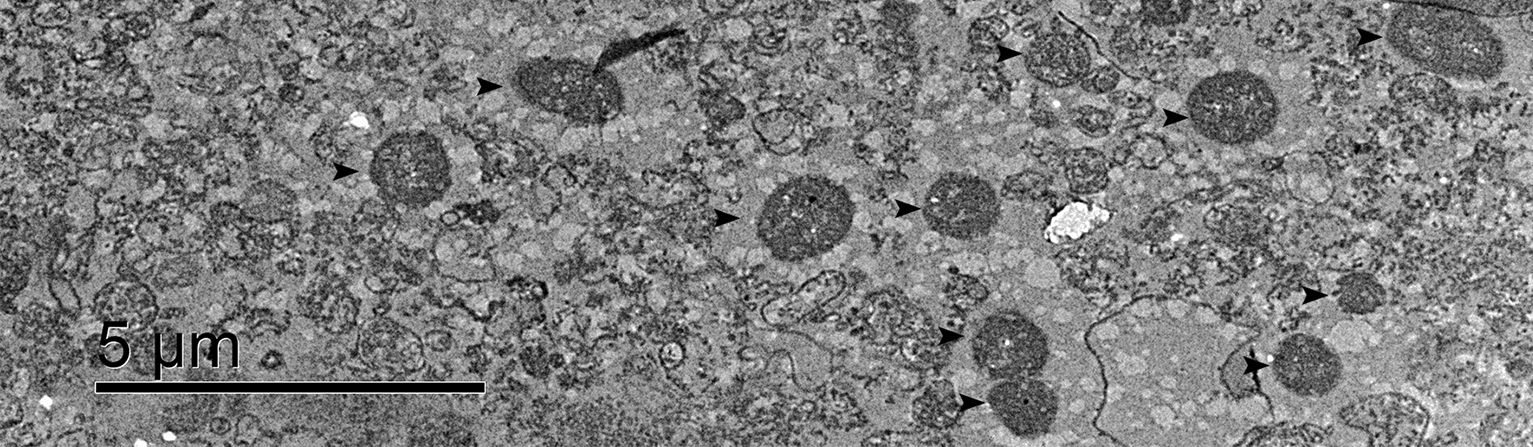
Observation of mitochondria with transmission electron microscopy. Isolated mitochondria were observed to be enriched; the black arrows indicate the intact mitochondria with high electron-density in the matrix. Bars, 5 μm

To further observe the morphology of the mitochondria, they were also examined by transmission electron microscopy (Fig. 4). We observed numerous mitochondria that contained a high electron density, indicating that the isolated mitochondria preserved a high viable activity [28]. All of these results suggested that the mitochondria were well enriched and that the structure remained intact, which allows for their use in further research.

### The mitochondrial membrane potential assay

**Fig. 5:**
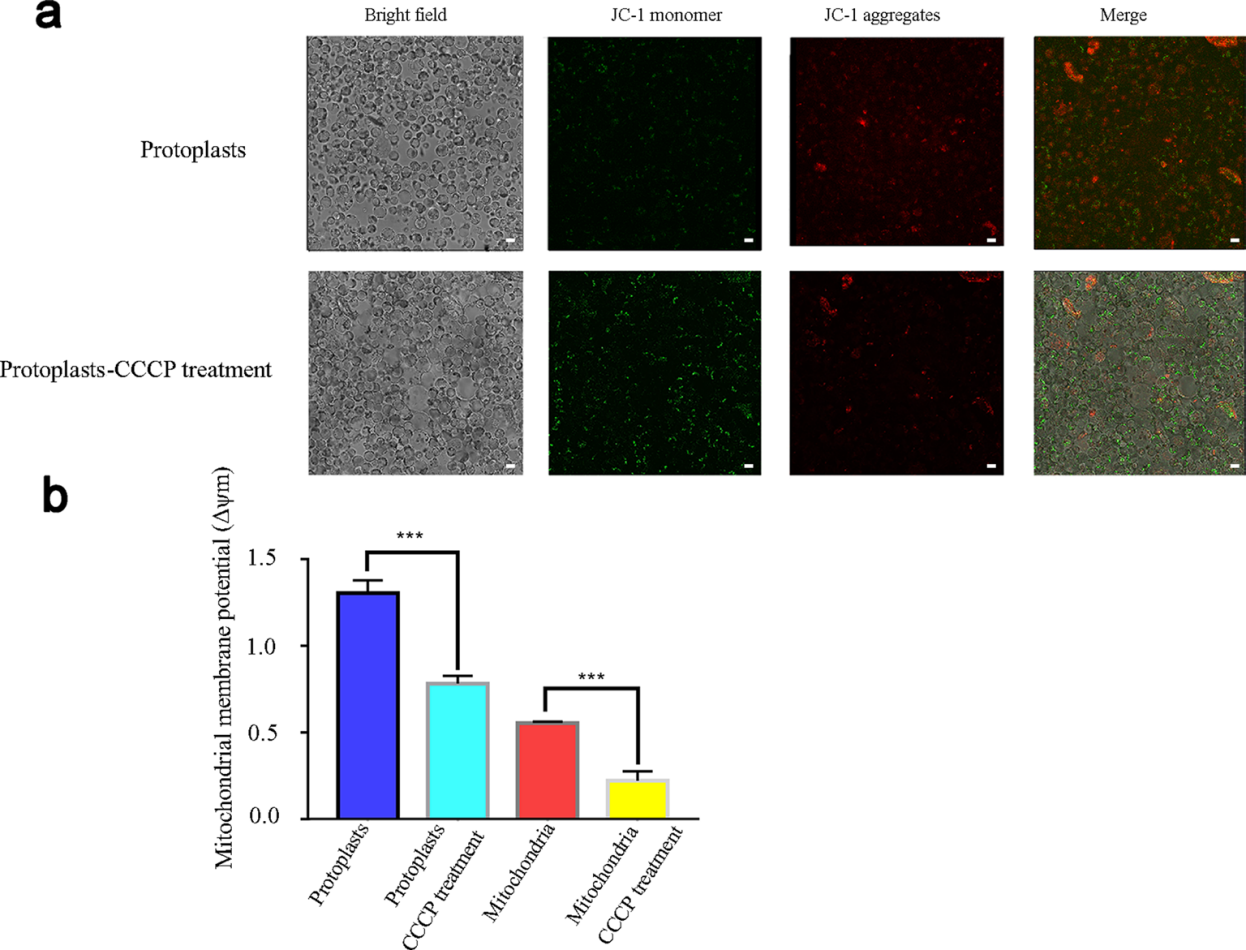
Examination of the mitochondrial membrane potential. **a** Examination of the mitochondrial membrane potential in protoplasts. JC-1 monomers exhibit a green signal, which indicates inactive mitochondria. JC-1 aggregates exhibit a red signal, which indicates viable mitochondria. Bars, 10 μΜ. **b** Examination of the mitochondrial membrane potential in isolated mitochondria. JC-1 monomers were detected by the wavelengths 514 nm/529 nm, and JC-1 aggregates were detected by the wavelengths 525 nm/590 nm. Error bars indicate the SDs of the means (n = 3). Significant differences were determined with the student’s t-test (***p < 0.005)

A previous study showed that fluorescence substrate JC-1 can specifically aggregate in the viable mitochondria and emit red fluorescence signals, while in apoptotic mitochondria, JC-1 exist in a monomeric state, which emits green fluorescence [29]. Therefore, JC-1 was then used to analyze the mitochondrial membrane potential. The rice protoplasts were incubated with JC-1 according to the protocol of the mitochondrial membrane potential detection kit. We also treated the protoplasts with carbonyl cyanide 3-chlorophenylhydrazone (CCCP), which is a mitochondrial membrane potential inhibitor, before staining with JC-1. The results showed that the protoplasts treated with CCCP exhibited stronger green signals than the untreated protoplasts (Fig. 5a), suggesting that more apoptotic mitochondria were generated when the protoplasts were treated with the mitochondrial membrane potential inhibitor.

Moreover, we used the Cytation 3 Multimode Reader to detect the fluorescence signal emitted by JC-1 aggregates or monomers, and the ratio of red and green fluorescence was calculated to indicate the mitochondrial membrane potential. As a control, the results demonstrated that CCCP-treated protoplasts showed a significantly decreased mitochondrial membrane potential compared to the untreated protoplasts. We then further assessed the membrane potential of isolated mitochondria, and the results showed that the membrane potential significantly declined, indicating that the isolated mitochondria kept its potential to some degree (Fig. 5b).

### The inner membrane complex activity of mitochondria

The activities of the ETC complex are very essential for the organism. Therefore, an activity assay of the mitochondrial inner membrane complex also needed to be performed in this study, even though the integrity of the mitochondria was confirmed.

**Fig. 6:**
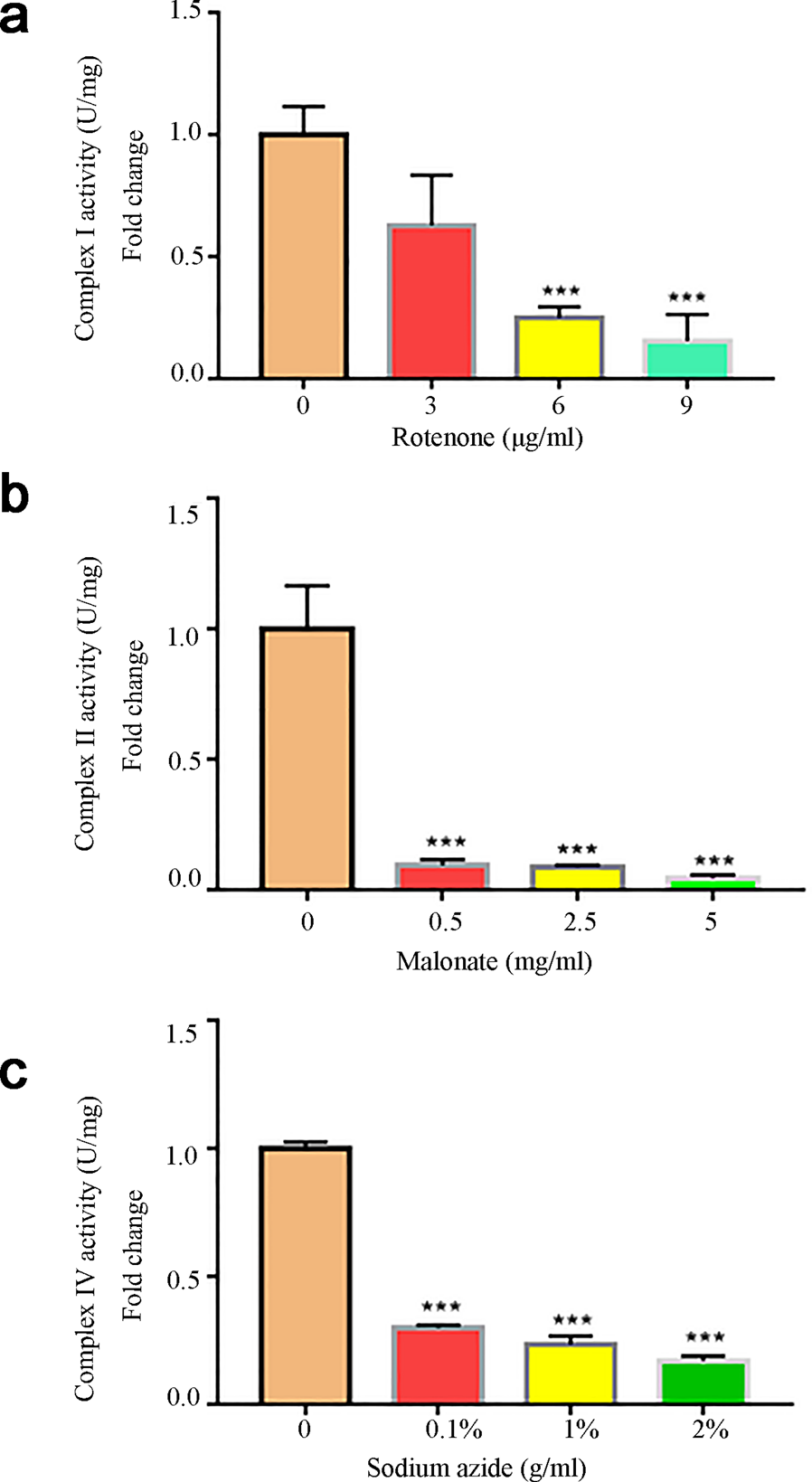
Mitochondrial inner membrane complex activity assay. The activities of complex I in panel (**a**), complex II in panel (**b**), and complex IV in panel (**c**) were tested by treatments with the inhibitors rotenone, malonate, and sodium azide, respectively. Error bars indicate the SDs of the means (n = 3). Significant differences were determined with the student’s t-test (***p < 0.005)

We investigated the activity of the isolated mitochondria using three different mitochondria inhibitors: rotenone (0 μg/ml, 3 μg/ml, 6 μg/ml, 9 μg/ml), which inhibits the ETC complex I, malonate (0 mg/ml, 0.5 mg/ml, 2.5 mg/ml, 5 mg/ml), which inhibits the ETC complex II, and sodium azide (0%, 0.1%, 1%, 2%), which acts on the ETC complex IV [30–32]. All three inhibitors were incubated with the extracted protoplasts for 30 min at 25 ℃ before isolating the mitochondria. The complex activity was assayed with the mitochondrial complex activity assay according to the manufacturer’s instructions with slight modification. The results showed that the activities of the mitochondrial inner membrane ETC complexes significantly declined under the different inhibitor treatments (Fig. 6). In addition, the complex activities were severely decreased with the increased concentration of inhibitor, which suggested that the isolated mitochondria could be used in subsequent studies.

## Discussion

### The simplified mitochondria isolation procedure

Plant mitochondria are difficult to isolate due to the presence of the cell wall, which is absent in the animal cell. The basic idea of our method was to obtain the protoplasts, which were similar to the animal cells, and then the mitochondria could be extracted easily according to the slightly modified mammalian mitochondrial isolation methods. We chose 10-day-old rice seedlings from which to isolate the protoplasts, since the plant cell undergoes active mitosis at this young stage and the cell wall can be easily degraded by cellulase and macerozyme. The cell structure of the extracted protoplast is similar to that of the animal cell, so it is reasonable to isolate the plant cell mitochondria by applying a homogenizer to burst the cell membrane in MIB. In this way, the mitochondria can be easily isolated from the seedlings. In addition, we also successfully obtained abundant rice protoplasts from the callus (Additional file 1: Fig. S2), suggesting that the callus can be used to isolate the rice mitochondria with this method.

Compared to the traditional rice mitochondria extraction methods that require strenuous labor to prepare the Percoll density gradient solution and multiple differential centrifugations, this improved method simplified the solution preparation by excluding the difficult preparation of a large amount of plant material and Percoll density gradient solution. Since most of the experimental time was spent in the enzyme solution incubation period, this method reduced the labor largely compared to the traditional mitochondria isolation method. The extraction of the mitochondria is not only important for the morphology and integrity of mitochondria but also essential for their activities and function. In this method, we greatly reduced the operation time to keep the mitochondria intact. In addition, the expenditure was also decreased since the expensive reagent Percoll and the ultra-speed centrifuge equipment are not necessary, which are not always affordable in many labs.

### Tips for a high yield of mitochondria

Based on our results, we believe that the successful acquisition of plant protoplasts is the guarantee for extraction. To harvest more mitochondria, the high yield of protoplasts is the key step. Although the chopped rice seedlings were suggested to be incubated with enzyme buffer for 5 h at 28 ℃ or overnight at 25 ℃, we recommend treating the young seedlings for 8 h at 25 ℃ (Table 1). For the callus, the incubation time can be extended to avoid the incomplete cell wall degradation. Generally, the shorter the incubation time, the higher the incubated temperature that is needed for the enzyme reaction. To yield more protoplasts from different tissues or species, various temperatures or times should be tested. The protoplasts are then lysed in MIB, and the incubation time is changeable when combined with the homogenization time. The longer the MIB incubation time, the less time for homogenization. Otherwise, both over-incubation and over-homogenization can result in broken mitochondria and inefficient mitochondria yields (Additional file 1: Fig. S3).

### The extracted mitochondria remained active

Since isolated mitochondria are vulnerable to damage and a fractured mitochondria structure greatly affects the results of experiments, in this study, we performed proteinase digestion experiments to examine the integrity of the mitochondrial structure. The mitochondrial marker proteins in the inner membrane and matrix were protected from digestion by proteinase K and thermolysin, which established that the isolated mitochondria and the outer membranes were intact. Furthermore, both of the CCCP-treated protoplasts and isolated mitochondria exhibited a significantly decreased mitochondrial membrane potential, which demonstrated that the mitochondria still kept their membrane potential in our extraction procedure.

We also examined the activity of ETC complexes in the inner membrane mitochondria. All of the tested mitochondrial ETC complexes in the inner membrane revealed a decline in activity, which exhibited a negative relationship with the increasing concentration of toxicant, indicating that the function of the mitochondrial ETC complexes was blocked by the toxicant. All of the results demonstrated that the isolated mitochondria could be used for biochemical analyses.

## Conclusions

In summary, the simplified method proposed in this study provides researchers an alternative way to isolate rice mitochondria. As the protoplasts of different plants are accessible [33–35], the mitochondria extraction method can be applied to other plants without preparing abundant plant materials. Compared to the traditional mitochondrial extraction methods, this mitochondria isolation method does not include expensive reagents, instruments and complicated steps. Moreover, the improved mitochondria extraction method can greatly shorten the time and save labor. All these improvements demonstrated that this simplified method has wide potential applications for plant mitochondria extraction for subsequent research.

## Materials and methods

### Plant materials

Two hundred rice seeds were prepared by dehulling the seeds with a sheller (Zhejiang Cheering Sewing Machine Company, China), followed by sterilization with 75% ethanol for 1–2 min. The rice was rinsed with sterilized water, followed by treatment with 0.15% HgCl_2_ for 15 min. The rice was cultivated on sterilized rooting medium for sprouting or induction medium for callus generation under dark conditions at 25 ℃ for 10 days and 20 days, respectively.

### Protocol for the mitochondria isolation

The improved plant mitochondria isolation method was based on protoplast extraction and lysis, and mitochondria were further isolated through differential centrifugation with a protocol that consisted of the following 10 steps:

- S1. Etiolated seedlings were collected, and the seeds were removed, after which the seedlings were dipped in balance buffer (0.6 M mannitol). For the callus, the newly generated calluses were chosen and dipped in balance buffer.
- S2. The middle region was selected, and the seedlings were chopped into 0.5 mm pieces with a razor blade, which were then put in balance buffer for at least 10 min.
- S3. Chopped seedlings or calluses were filtered with a nylon net (300 mesh, 0.05 mm aperture) and transferred into enzyme buffer (10 mM MES, pH 5.7, 0.6 M mannitol, 0.1% BSA, 1.5% (w/v) cellulase R10 (YakultHon sha, Tokyo, Japan), 0.75% (w/v) macerozyme R10 (Yakult Honsha), 1 mM CaCl_2_, 1 mM β-mercaptoethanol, 50 μg/ml carbenicillin) in a water bath for 10 min at 55 ℃, after which the mixture was allowed to cool to room temperature.
- S4. The mixture was shaken at 80 rpm for 5 hours at 28 ℃ in a dark environment.
- S5. Chopped seedlings or calluses were filtered, and the filtrates were collected in a 7 ml EP tube and centrifuged at 600 g for 3 min at 25 ℃.
- S6. The pellet was resuspended with W5 solution (2 mM MES, pH 5.8, 154 mM NaCl, 125 mM CaCl_2_, 5 mM KCl, 5 mM glucose) and centrifuged at 600 g for 3 min at 25 ℃. The supernatant was discarded, and this step was repeated.
- S7. The pellet was resuspended with MIB (70 mM sucrose, 210 mM mannitol, 10 mM HEPES, 1 mM EDTA, 1 mM PMSF, pH 7.5) and shaken for 1 hour at 4 ℃ gently.
- S8. The suspension was homogenized using a Teflon dounce homogenizer, and the homogenization time for the sufficient lysis of the cell had to be determined, as over-lysis results in burst mitochondria. Empirically, 20 strokes with the homogenizer were sufficient to burst the protoplast.
- S9. The mixture was centrifuged at 600 g for 10 min at 4 ℃, and the supernatant was transferred into new EP tube and centrifuged at 11,000 g for 10 min at 4 ℃.
- S10. The supernatant was discarded, and the mitochondria were resuspended in storage solution (0.4 M mannitol, 50 mM Tris, 1 mM EDTA, 5 mM KCl, 5% DMSO, pH 7.4).

### Mitochondrial purity determination

Different components of the cell were prepared, including total protoplast, nucleus, mitochondria and the cytoplasm, which were resuspended in lysis buffer (100 mM Tris–HCl, pH 8.0, 150 mM NaCl, 5 mM EGTA, 5 mM EDTA, 10 mM DTT, 0.5% [v/v] Triton X-100). The samples were mixed with loading buffer (250 mM Tris–HCL, 10% SDS, 0.5% BPB, 50% glycerol, 5% 2-ME) and were denatured at 98 ℃ for 10 min. The proteins were separated by a 10% SDS-page gel. For the western blot, the proteins were transferred onto a PVDF membrane (Bio-Rad, Hercules, CA, USA). Western blots were conducted by using the following primary antibodies and dilutions: anti-rubisco 1:5000, anti-VDAC 1:2000, anti-histone 1:5000, anti-COX II, 1:1000 and anti-catalase 1:1000. This was followed by incubation with the corresponding secondary antibody at a 1:5000 dilution. Signals were visualized by chemiluminescence (Bio-Rad).

For the genome purity assessment, the protoplasts and mitochondria were resuspended in CTAB buffer (2% CTAB, 20 mM Na_2_EDTA, 100 mM Tris–HCl, 1.4 M NaCl, pH 8.0) at 60 ℃ for 1 h. The DNA was extracted according to the standard protocol. The DNA concentration was measured by a Nanodrop 2000c and diluted to 15 ng/ul. The PCR reaction was performed with 2 × Taq plus master mix (Vazyme Biotech Co, P111/P112, Nanjing, China) according to the manufacturer’s instructions. The forward primer *actin*-F (5′-AACTGAAACCCCCATGTCCC-3′) and reverse primer *actin*-R (5′-TGCAGAACGGAAAAGTCCCA-3′) were used for the amplification of the *actin* domain (*actin*, LOC_Os03g61970). The forward primer *cox III*-F (5′-GACAAATGGGAATAACCGAA-3′) and reverse primer *cox III*-R (5′-GGGGAAGGAAAAACGAGCAG-3′) were used for the amplification of the *cox III* domain (*cox III*, LOC_Osm1g00110.1). The forward primer *rubisco*-F (5′-ATGTCACCACAAACAGAAAC-3′) and reverse primer *rubisco*-R (5′- CTAGCTATCTAGTTTATCTA-3′) were used for the amplification of the *rubisco* large subunit domain (*rubisco*, OrsajCp033). The PCR conditions were as follows: 95 °C for 5 min, followed by 35 cycles of 95 °C for 15 s, 55 °C for 15 s, and 72 °C for 30 s. The PCR products were separated in a 2% agarose gel and visualized by a gel imaging system (GE).

### Intact mitochondria examination

The extracted mitochondria were incubated with proteinase K (0 μg/ml, 5 μg/ml, 20 μg/ml, 50 μg/ml) or thermolysin (0 μg/ml, 10 μg/ml, 50 μg/ml, 100 μg/ml) at 4 ℃ for 20 min. CaCl_2_ was added to the thermolysin to a final concentration of 0.5 μM. All of the reactions were quenched by adding EDTA to a final concentration of 5 mM, after which SDS-page electrophoresis was performed, and western blotting was conducted as described above.

### Transmission electron microscopy observation

The sample preparation of mitochondria for transmission electron microscopy is described as follows: (i) the isolated mitochondria were fixed in 2% glutaraldehyde (2.5% glutaraldehyde, 100 mM NaH_2_PO_4_H_2_O, 80 mM Na_2_HPO_4_12H_2_O) overnight and washed in phosphate buffer (100 mM NaH_2_PO_4_H_2_O, 80 mM Na_2_HPO_4_12H_2_O) 5 times for 20 min each; (ii) the sample was then fixed with 1% osmic acid (1% osmic acid, 100 mM NaH_2_PO_4_H_2_O, 80 mM Na_2_HPO_4_12H_2_O) for 2 h, followed by 5 washes in phosphate buffer for 30 min each; (iii) the sample was dehydrated in ethanol at increasing concentrations of 15%, 30%, 50%, 70%, 80%, 85%, 90%, and 95% successively, 20 min each; (iv) the sample was dehydrated in ethanol 2 times for 45 min each; (v) the sample was treated with a mix of ethanol and propylene oxide (the ratio was 1:1) for 30 min; (vi) the sample was treated with propylene oxide and spur resin (the ratios were 3:1, 1:1, and 1:3, with each different ratio used for 12 h in that order); (vii) the sample was embedded in spur resin and incubated in an oven for 8 h at 40℃, followed 60℃ for 1–2 days; (viii) the sample was sliced with an ultrathin microtome, followed by staining, washing, and drying; (ix) the nickel net that was loaded with the sample was observed with a JEM-1400 Plus Transmission Electron Microscope.

### Mitochondrial membrane potential analysis

For the protoplasts, the suspension was centrifuged at 600 g for 3 min, and the pellets were resuspended in W5 solution and diluted to an appropriate concentration (to ensure no cell stacking in the light microscopy). The protoplast was incubated with 1X JC-1 staining solution (6,6′-tetrachloro-1, 1′, 3,3′-tetraethylbenzimidazole-carbocyanide iodine) (1:50) (Beyotime) for 20 min, following 20 min CCCP (carbonyl cyanide m-chlorophenylhydrazone) treatment. The dual emission fluorescence was detected by fluorescence microscopy (FV1000 confocal system). Briefly, the JC-1 monomer was detected using a 514 nm excitation wavelength and a 529 nm emission wavelength. JC-1 aggregates were detected using a 525 nm excitation wavelength and a 590 nm emission wavelength. For the fluorescent enzyme labeling detection, three replicates were used for each sample, which were loaded into a black enzyme plate (Costar) for dual fluorescence detection by a mono-photometric system (Cytation 3 multimode reader, PengDe).

For the mitochondria, the pellets were resuspended in storage buffer and treated with CCCP for 5 min, followed by incubation with 0.1X JC-1 staining solution for 5 min. The dual fluorescence was detected by a mono-photometric system, similar to the protoplasts.

### Mitochondrial complex activity detection

The activities of mitochondrial complex Ι, complex II, and complex IV were detected by a Nanodrop 2000c using the MRC (mitochondrial respiration chain) complex Ι, complex II and complex IV activity assay kit (Solarbio Life Sciences, Beijing, China). Briefly, the protoplasts were treated with rotenone (0 μg/ml, 3 μg/ml, 6 μg/ml, 9 μg/ml), malonate (0 mg/ml, 0.5 mg/ml, 2.5 mg/ml, 5 mg/ml) and sodium azide (0%, 0.1%, 1%, 2%) for 30 min at 25 ℃, respectively. Then, the mitochondria isolation procedure followed, as outlined above. All of the assays were performed according to the manufacturer’s instructions.

## Supplementary information

**Additional file 1: Figure S1. ** Digestion pattern of thermolysin treated mitochondira. The isolated mitochondria were digested with an increasing thermolysin concentration. VDAC and cytochrome C oxidase II (COXII) were examined as the marker proteins of the mitochondrial outer membrane and inner membrane, respectively. **Figure S2.** Protoplasts isolated from calli. **a** The calli were cultured on the subculture medium. **b** The protoplasts were isolated from the rice callus, which showed the complete degradation of cell wall and the spherical cell shape. Bar, 10 μΜ. **Figure S3.** Over-lysis of the mitochondria. Over homogenization of the protoplast suspension can cause the mitochondria burst. Black arrows indicate the broken mitochondria membrane, white arrows indicate the inner membrane cristae of intact mitochondria. Bar, 500 nm.

## Data Availability

All the data generated or analyzed during this study are included within this article.

## Abbreviations

ETC: Electron transfer chain
MIB: Mitochondrial isolation buffer
FLB: Full lysis buffer
VDAC: Voltage-dependent anion channels
COX II: Cytochrome C oxidase II
NAD3: NADH dehydrogenase subunit 3
CCCP: Carbonyl cyanide 3-chlorophenylhydrazone
