# Supplementary material for: A simplified method to isolate rice mitochondria
Source: Plant Methods. 2020 Nov 4;16:149. doi: 10.1186/s13007-020-00690-6 (PMC7640673; doi:10.1186/s13007-020-00690-6)
Supplement: Supplementary file 1 — Additional file 1: Figure S1. Digestion pattern of thermolysin treated mitochondira. The isolated mitochondria were digested with an increasing thermolysin concentration. VDAC and cytochrome C oxidase II (COXII) were examined as the marker proteins of the mitochondrial outer membrane and inner membrane, respectively. Figure S2. Protoplasts isolated from calli. a The calli were cultured on the subculture medium. b The protoplasts were isolated from the rice callus, which showed the complete degradation of cell wall and the spherical cell shape. Bar, 10 μΜ. Figure S3. Over-lysis of the mitochondria. Over homogenization of the protoplast suspension can cause the mitochondria burst. Black arrows indicate the broken mitochondria membrane, white arrows indicate the inner membrane cristae of intact mitochondria. Bar, 500 nm. [file 13007_2020_690_MOESM1_ESM.doc]

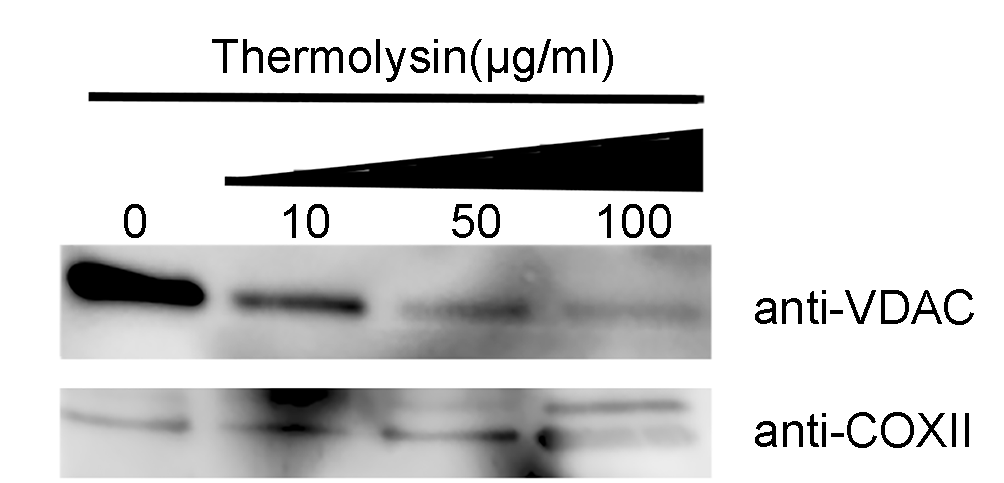


Figure S1. Digestion pattern of thermolysin treated mitochondira. The isolated mitochondria were digested with an increasing thermolysin concentration. VDAC and cytochrome C oxidase II (COXII) were examined as the marker proteins of the mitochondrial outer membrane and inner membrane, respectively.


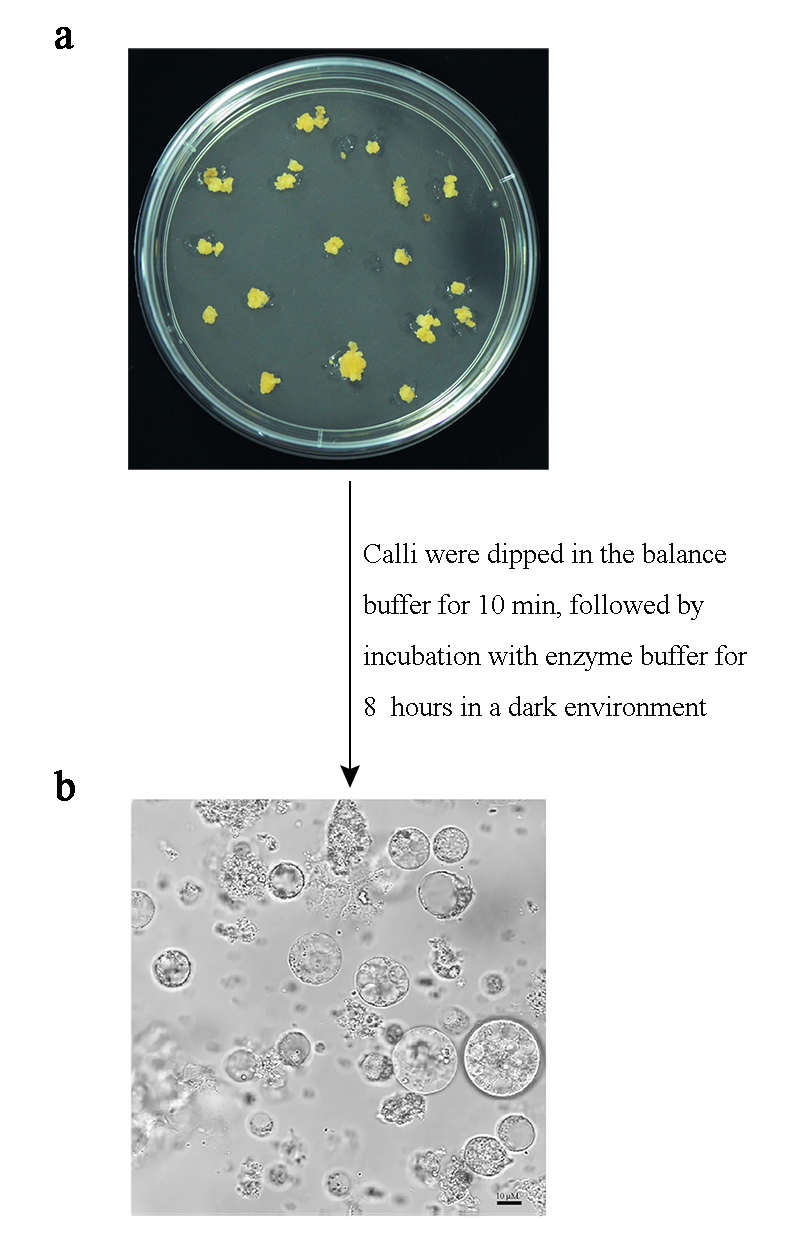


Figure S2. Protoplasts isolated from calli. **a** The calli were cultured on the subculture medium. **b** The protoplasts were isolated from the rice callus, which showed the complete degradation of cell wall and the spherical cell shape. Bar, 10 μΜ.


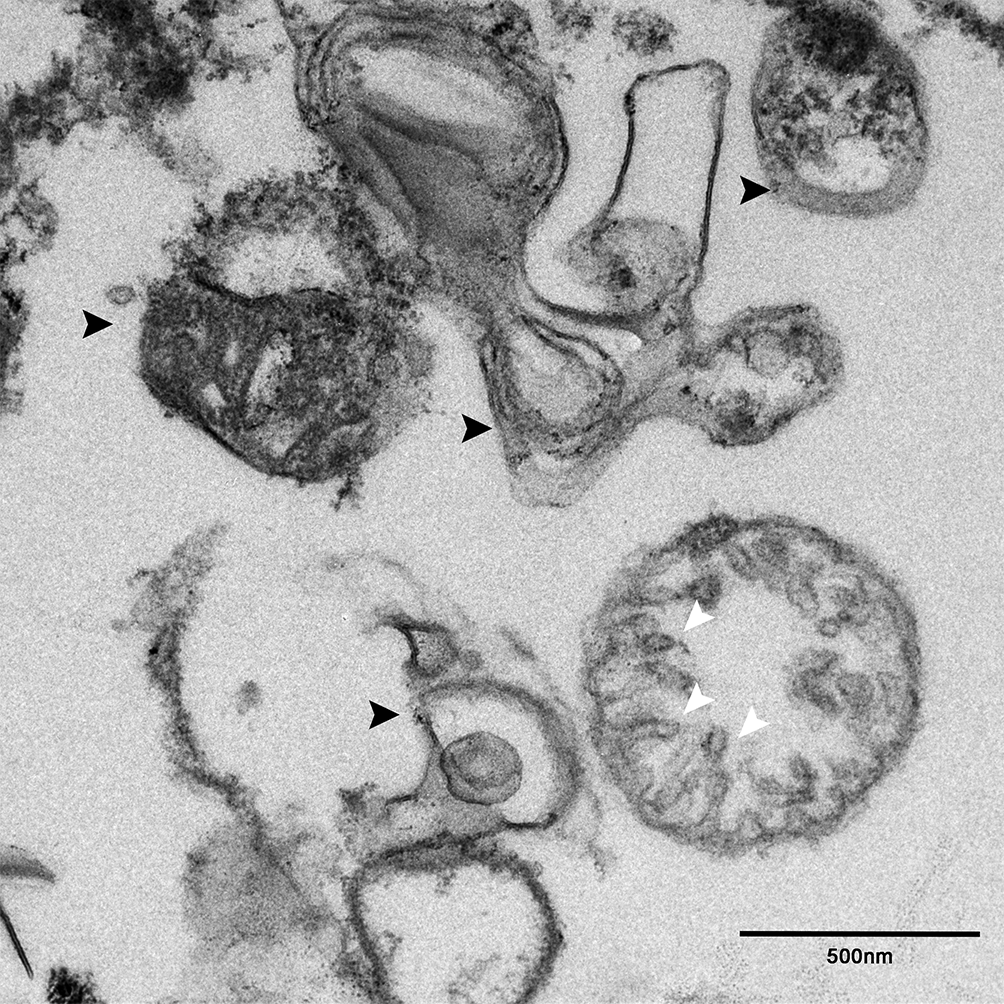


Figure S3. Over-lysis of the mitochondria. Over homogenization of the protoplast suspension can cause the mitochondria burst. Black arrows indicate the broken mitochondria membrane, white arrows indicate the inner membrane cristae of intact mitochondria. Bar, 500 nm.
